# A Comparative Study of the Stress Distribution in Different EndodonticPost-RetainedTeeth with and without Ferrule Design—A Finite Element Analysis

**DOI:** 10.5402/2011/102329

**Published:** 2011-06-14

**Authors:** Lokanath Garhnayak, Hari Parkash, D. K. Sehgal, Veena Jain, Mirna Garhnayak

**Affiliations:** ^1^Department of Prosthodontics, Rajasthan Dental College and Hospital, Jaipur 302026, India; ^2^Department of Prosthodontics, Centre for Dental Education and Research, All India Institute of Medical Sciences, New Delhi 110029, India; ^3^Department of Applied Mechanics, Indian Institute of Technology, New Delhi 110029, India

## Abstract

*Purpose*. To analyze the stress distribution in an endodontically treated maxillary central incisor restored with various post-core systems and assess the benefit of ferrule using finite element analysis. *Material and Methods*. Twelve models with metal ceramic crown were created based on the combination of three types of post-core systems (titanium post-composite resin core, nickel-chromium post-core, and fiber reinforced composite resin post-composite resin core), two varieties of posts (tapered, parallel), and with or without ferrule. 100 N load was applied in three directions and the von Mises stress was compared. *Results*. Ferrule made no difference in stress distribution for the titanium and nickel-chromium posts, though it showed some stress reduction in fiber-reinforced composite resin posts. Nickel-chromium cast post-core transmitted the least amount of stresses to the dentin despite producing the maximum stress. *Conclusion*. Incorporation of ferrule offered some degree of stress reduction in nonmetal post, and it increased the stresses within cervical dentin.

## 1. Introduction

The restoration of endodontically treated teeth has been a concern for prosthodontists for more than 100 years [1]. The increasing effectiveness and predictability of endodontic therapy have only made the challenge more evident. If a root canal treated tooth is severely damaged beyond the realms of restoration by conventional means, the clinician is left with no alternative other than a post-core. The predominant function of an endodontic post is to retain the core [2], which subsequently provides a suitable foundation for final restoration. The selection of a particular type of endodontic post is based on its mechanical properties, ease of fabrication, biocompatibility, availability in the market, and the cost factor. Custom made nickel-chromium (Ni-Cr) cast post-core system is routinely used because of its precision and ease of fabrication in the laboratory. Among the prefabricated posts, titanium (Ti) posts are very popular owing to their proven biocompatibility. Newer post materials like ceramics and fiber reinforced composite resin (FRC) [3–5] are also gaining popularity due to their unique esthetic properties. The ability of post-core system to sustain masticatory forces and remain firmly seated in the tooth is essential for the survival of a restoration. One other important design consideration in the restoration of an endodontically treated tooth is the ferrule. This has been described as a metal band that encircles the external dimension of the residual tooth [6]. A properly built ferrule significantly reduces the incidence of fracture in the nonvital tooth by reinforcing the tooth at its external surface [7, 8]. It resists the lateral forces from the tapered dowels and the leverage from the crown in function [6]. It also increases the retention and resistance of the restoration [6]. Combined together, the pattern of stress distribution by the endodontic posts and the ferrule design under masticatory load is of great importance in ensuring an optimal design for the prosthesis.

Stress analysis in dentistry has been a topic of interest for the past few decades. Traditional methods of experimental stress analysis includes mechanical stress analysis [9], photoelastic method [10], and electrical strain gauge [11] and so forth. Among these, the photoelastic method was favored due to its ability to incorporate irregular geometry and the ready visualization of stress concentration in the materials under loads [12]. However, the exact duplication of material properties of the model was difficult, and hence the actual stresses in the real case was only approximated. An approach to stress analysis of dental structures which deals with the previously described complexities while avoiding the shortcomings of photoelastic analysis is the “finite element method” (FEM). Finite element method is a numerical tool, which is popularly used to analyze very complex and irregular structures [13]. Since its beginning in 1956, the versatility and efficiency of this method has been recognized in various engineering fields like civil, mechanical, and aeronautical engineering [13]. Besides this, it has a wide application in biomechanical sciences like dentistry and orthopedics.

This study is an attempt to use FEM to predict, analyze, and compare stress distribution of different types of post-core systems with and without ferrule in metal ceramic crown restored endodontically treated maxillary central incisor.

## 2. Material and Methods

The study was conducted in the Department of Dental Surgery, All India Institute of Medical Sciences, New Delhi and the Department of Applied Mechanics, Indian Institute of Technology, New Delhi, India. 

### 2.1. Finite Element Modeling

**Figure 1:**
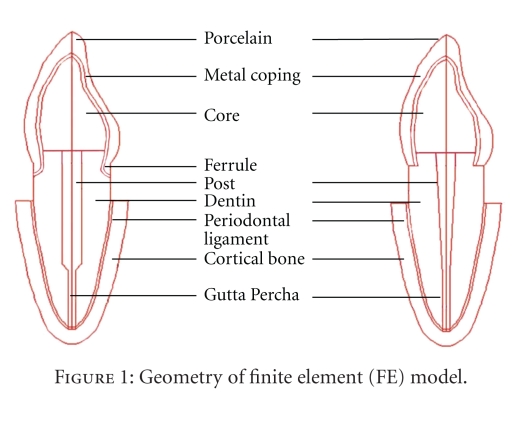
Geometry of finite element (FE) model.

**Figure 2:**
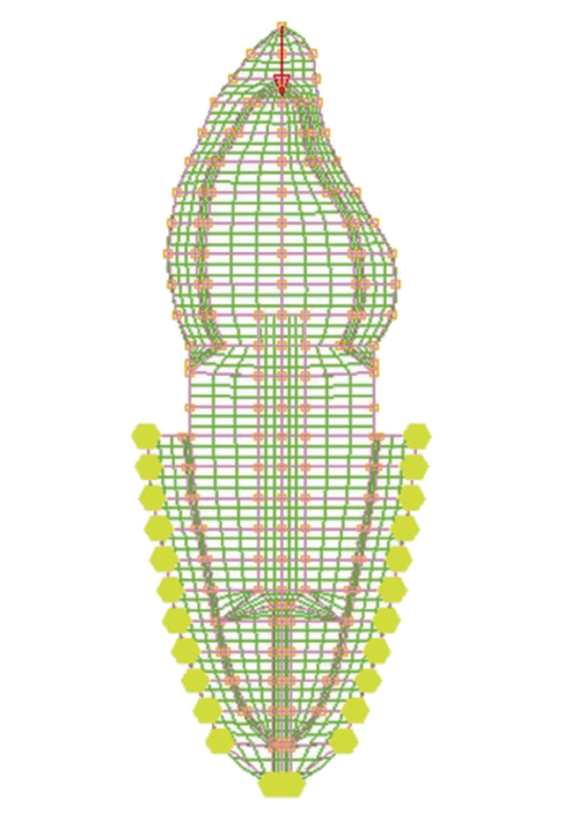
Mesh generated over ferrule model.

**Figure 3:**
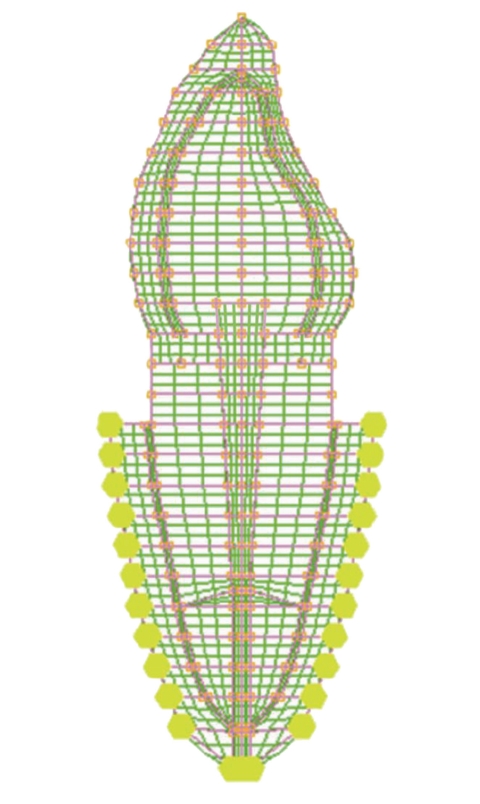
Mesh generated over nonferrule model.

The dimensions of various parts of the model for maxillary central incisor (Bucco-lingual view) were adopted from standard dental literature (Table 1) [6, 14–17]. The finite element model geometry (Figure 1) was generated on the computer screen by provision of various entities such as grids, lines and patches, and so forth. Each model was divided into small elements interconnected at nodes and a finite element mesh was superimposed on the model (Figures 2 and 3). The elements used in this analysis were four noded quadrilateral elements and three noded triangular elements. In total, 12 different, 2-dimensional plane strain models were generated for different variety of post-core, with and without ferrule design. 

The different parts of these models are illustrated in Figure 1. Three different post materials—titanium (Ti), nickel-chromium (Ni-Cr), and fiber reinforced composite resin (FRC) were evaluated in tapered and parallel forms. The length of the post was kept at 10.5 mm for both the forms. The width of the parallel post was 1.5 mm throughout its length and that of the tapered design decreased from 1.5 mm at the cervical portion to 0.6 mm at the apical portion (Table 1). While the core material for custom made Ni-Cr cast post-core was the same alloy, that of titanium and fiber reinforced composite resin posts, was composite resin. The height of core was 6.5 mm built over 2 mm of residual dentin coronally from crown margin (Table 1). The artificial crown in all cases was metal ceramic crown, and the total height of crown was 10.5 mm with an additional 1.5 mm cervical extension in the ferrule model (Figure 1, Table 1). The thickness of the crown margin at the cervical portion and at the incisal edge was 1.2 mm and 2.0 mm, respectively. The thickness of metal coping (Ni-Cr) was modeled to be 0.3 mm (Table 1). 

The properties of various materials like modulus of elasticity (“*E*”) and Poisson's ratio (“*v*”), adopted from the standard literature (Table 2) [5, 18–23], were applied for the respective materials the finite element model. The displacement boundary conditions were fixed at the alveolar bone trough surrounding the root.

### 2.2. The Loading Condition and Analysis

A static load of 100 N was planned to be applied to the maxillary central incisor tooth restored with post, core, and crown. Since the model in the current study was 2-dimensional (plane strain model with 1 mm thickness) and the mesiodistal diameter of maxillary central incisor was 8.5 mm, the force applied to the FE model was calculated as total force/mesiodistal width of tooth, that is, 100 N/8.5 = 11.76 N (approximated to 12 N).

Each model was evaluated under forces directed in three different directions, (1) vertical—at the incisal edge of the crown, (2) oblique—at 45° and at a distance of 2 mm from the incisal edge on the lingual aspect of crown, and (3) horizontal—at the same point like oblique force. The stress pattern and maximum value of the generated von Mises stress were used to interpret the results of the study as it indicates the site where yielding of ductile material is likely to occur.

All the modeling, meshing, and the analysis were performed in NISA (numerically integrated element for system analysis).

## 3. Results

Under vertical load, the overall stress produced within tapered posts were more for Ni-Cr cast post-core (4.470 MPa) and Ti post with composite resin core (3.489 MPa; Table 3). The FRC tapered and parallel posts produced approximately the same stress values (0.702 MPa) within the post as well as in the adjacent dentin (Table 3). 

Common to all the type of post-core systems, when the direction of load was changed from vertical to horizontal, the stress levels increased as the forces became more oblique (Table 4) and finally reached the highest levels when they were absolutely horizontal (Table 5). For the horizontal loading, the Ni-Cr cast post-core produced the maximum stresses (16.99 MPa) within the post-core system. Interestingly, it was the same system that also transmitted the least of stresses to the surrounding dentin. Although FRC posts recorded the lowest stress level within the posts, the stresses transmitted to the surrounding dentin were more (25.77 MPa, Table 6). Ferrule did not reduce the stress values either in the tapered or parallel forms of Ti and Ni-Cr posts. However, there was some reduction in stress levels within the tapered and parallel FRC posts under oblique and horizontal load. Incorporation of ferrule was found to increase the magnitude of the stresses in the cervical dentin as given in Table 6.

## 4. Discussion

**Figure 4:**
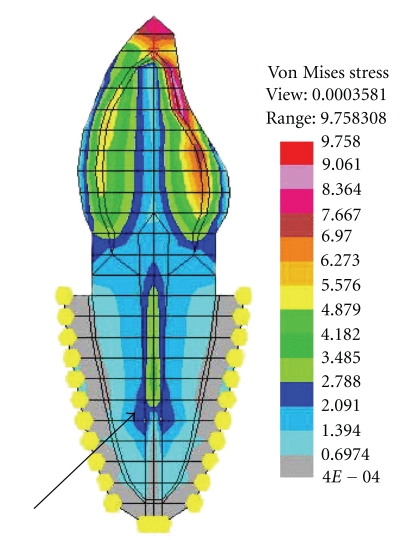
“Wedging effect” with tapered post.

**Figure 5:**
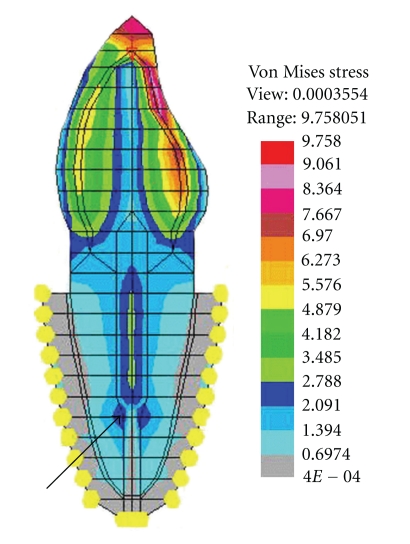
Poorly defined “wedging effect” with parallel post.

Stress plots (von Mises) under vertical load indicated that the overall stress produced by tapered post was more as compared to parallel post for both ferrule and nonferrule group, in case of Ti and Ni-Cr posts. The difference in the stress values were better appreciable in the middle and clearly defined in the apical one-third of the post. This exemplifies the “wedging effect” (Figure 4) seen in the dentin adjacent to the apex of the tapered post, a finding consistent with the existing literature [24, 25]. The “wedging effect” is due to the reduction in the dimensions of the tapered post near the apical portion. This results in the same amount of load distributed over a smaller area as compared to the parallel post (Figure 5). However, no such difference was observed for FRC posts in case of both ferrule and nonferrule group, a finding that can be explained on the basis of difference in the modulus of elasticity (“*E*” value). Since, the “*E*” value of Ti and Ni-Cr are higher than the dentin, stresses are mainly concentrated within the post and in the adjacent dentin. But, the difference in “*E*” values between dentin and FRC was minimal. Thus, there is approximately equal distribution of stresses within the post as well as in the dentin in case of FRC post. The finding that the “wedging effect” was more pronounced in case of Ni-Cr as compared to Ti, suggests a direct relationship between “*E*” value of the material and the intensity of “wedging effect.”

A common observation noticed for all 3 types of materials that is, Ti, Ni-Cr, and FRC was that stress values were maximum under horizontal load, followed by oblique, and the least for vertical load. This could be due to the higher effect of leverage that occurs with oblique and horizontal loads. 

**Figure 6:**
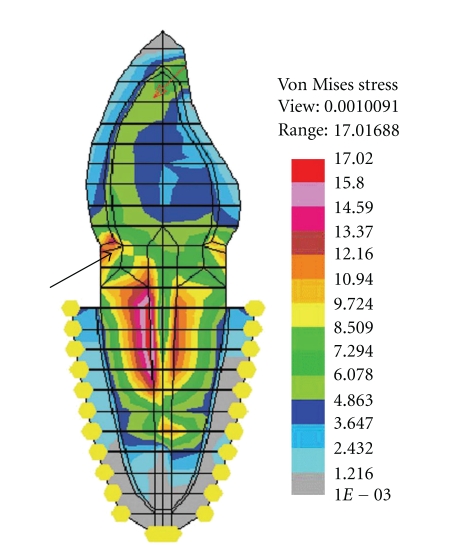
Increased stresses (von Mises) in cervical dentin with ferrule design.

**Figure 7:**
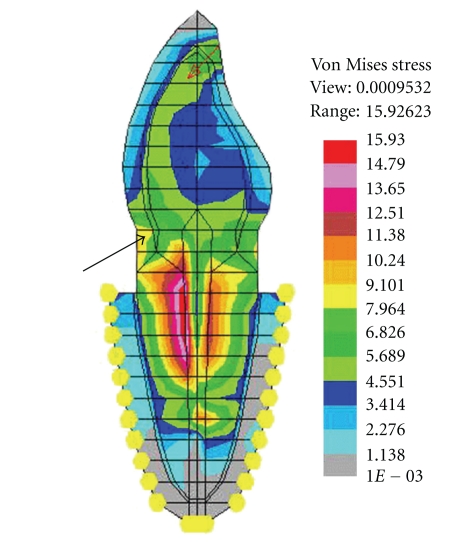
Reduced stresses (von Mises) in cervical dentin without ferrule design.

There is mixed opinion regarding the efficacy of ferrule in increasing the threshold of failure load in an endodontically treated tooth. Some mechanical studies favor the placement of ferrule as it confers increased fracture resistance to the endodontically treated teeth. Libman and Nicholls [26] have reported that the preliminary failure occurred with lower number of cyclic load (4.0 kg) in endodontically treated central incisors with ferrule length of 0.5 and 1.0 mm as compared to 1.5 and 2.0 mm.EvenIsidor et al.[7] have observed an increased fracture resistance of endodontically treated tooth to cyclic loading with increasing ferrule length.Barkhordar et al.[8] reported that a metal collar of approximately 3° significantly enhanced the resistance to root fracture of endodontically treated maxillary central incisor. In contrast, Tjan and Whang [27] found that addition of a metal collar did not enhance the resistance to root fracture. Al-Hazaimeh and Gutteridge [28] demonstrated that the additional use of a ferrule (2 mm) preparation had no benefits in terms of resistance to fracture when composite cement and core materials were utilized with a prefabricated parapost system. The results drawn from the present study, suggested that the placement of ferrule at the cervical portion of the root reduced the stresses in case of FRC post with composite resin core only and the stress reduction was greater for horizontal load than the oblique load. Probably, this could be due to the use of ferrule with nonmetal post-core concentrating more stresses in and around the metal collar (because of higher “*E*” value) in the cervical dentin and transmitting less stresses to the underlying FRC post. Placing an additional metal collar did not have much significance in stress reduction within metal post, that is, Ni-Cr, and Ti. To the contrary, the metal collar increased the stresses in the cervical dentin around it (Figures 6 and 7). The lack of benefit of ferrule placement when Ti post with composite resin core and Ni-Cr cast post-core were used could be explained on the basis of their higher modulus of elasticity attracting greater stresses within the post. This is in agreement with the results of the photoelastic study conducted byLoney et al.[29] who reported that the metal collar (1.5 mm) produced higher stress value at the cervical and apical regions of post. The FEM study ofIchim et al.[30] revealed that the ferrule increased the mechanical resistance of a post/core/crown restoration in central incisor. But, it also created a larger area of palatal dentin under tensile stress, a condition favorable for a crack development on the palatal aspect of the root eventually leading to an oblique root fracture. In comparison, a restoration without ferrule was prone to fail primarily by debonding and subsequently by root fracture through the lever action of the loose post. 

Ni-Cr cast metal post-core recorded minimal stress levels in the surrounding tissue as they were concentrated along the Ni-Cr cast post-core owing to its higher “*E*” value. Eskitaşcioğlu et al. [31] found that the cast post-core accumulated higher stresses within the post-core system and transmitted lower stresses to supportive structures whereas fiber composite laminate (FCL) post-core produced less stresses within post-core system and transmitted greater stresses to the supportive structures. When stress patterns were compared between core materials with different posts, it was observed that the overall maximum stress values were produced with Ni-Cr. 

From the analysis of the stress plots obtained from various FE models, Ni-Cr cast post-core appears to be most advantageous, since it transmitted less stresses to the supportive structures. Alhough the stresses produced within the FRC post (with composite resin core) were less as compared to Ni-Cr post, the former transmitted greater stresses to surrounding dentin mainly in oblique and horizontal load directions.Martínez-Insua et al.[3] reported that although the FRC posts (“*E*” value 15 GPa) fail at lower loads than stainless steel (“*E*” value 200.0 GPa), the failure occurred at loads greater than those occurring in the mouth. The failure was also not catastrophic as debonding occurs between individual fibers and the matrix before frank fracture of the FRC post. This can be considered as an advantage with FRC post because the reverse situation generally necessitates extraction of the tooth. Pegoretti et al. [32] reported that the incorporation of glass fiber in FRC, instead of carbon fiber, resulted in the lowest level of peak stresses to the surrounding dentin by virtue of its stiffness being much similar to the latter. Taking all these factors into consideration, FRC posts appear promising for the long-term success of endodontically treated teeth [3, 32].

## 5. Conclusions

1. Under vertical load, the overall stresses produced within the tapered posts were more for Ni-Cr cast post-core and Ti post with composite resin core. The FRC tapered and parallel posts produced approximately the same stress levels within the posts as well as in the adjacent dentin.
2. For all the models evaluated, the maximum stresses in the posts and surrounding structures were recorded with horizontal load, followed by oblique and vertical loads.
3. Ni-Cr cast post-core produced maximum stresses within the post-core system and the least amount of stresses transmitted to the surrounding dentin. Although FRC posts recorded minimal stress level within the posts, the stresses transmitted to the surrounding dentin were more as compared to Ni-Cr cast post-core.
4. Ferrule did not reduce the stress values either in the tapered or parallel posts of Ti and Ni- Cr. However, there was some degree of stress reduction within the tapered and parallel FRC posts under oblique and horizontal load.
5. Incorporation of ferrule increased the stresses in the cervical dentin in all the types of post-core systems evaluated in the study.

## Figures and Tables

**Table 1:** Dimensions of structures in FE model [6, 14–17].

No.	Different parts in FE model	Dimensions (mm)
(1)	Tooth [14]	
	(a) Root length	13.0 mm
	(b) Root diameter	6.0 mm (cervical)
(2)	Periodontal ligament [15]	0.2 mm (width)
(3)	Cortical bone [16]	1.2 mm (thickness)
(4)	Gutta Percha [17]	4.5 mm (length)
(5)	Endodontic post [17]	
	Length	10.5 mm
	Diameter	1.5 mm (parallel post)
		0.6–1.5 mm (tapered post)
		10.5 mm (height)
(6)	Crown [17]	1.2 mm (thickness at cervical margin)
		2.0 mm (thickness at incisal edge)
(7)	Core [17]	6.5 mm (height) + 2 mm residual dentin
(8)	Ferrule design [6]	1.5 mm (length)

**Table 2:** Material properties in FE model [5, 18–23].

No.	Material	Modulus of elasticity (“*E*”) (GPa)	Poisson's ratio (“*v*”)
(1)	Dentin [18]	18.6	0.31
(2)	Periodontal ligament [19, 20]	0.0000689	0.45
(3)	Cortical bone [21]	13.7	0.30
(4)	Gutta Percha [19, 20]	0.069	0.45
(5)	Titanium [22]	120.0	0.30
(6)	Ni-Cr alloy [22]	203.6	0.30
(7)	Fiber reinforced composite resin [5]	15.0	0.28
(8)	Composite resin [20]	8.3	0.28
(9)	Porcelain [23]	69.0	0.28

**Table 3:** Comparison of stress* distribution between tapered and parallel posts of different materials with vertical load.

Materials	Region^†^	Tapered post		Parallel post	
		Ferrule	Nonferrule	Ferrule	Nonferrule
Ti	A	2.094	2.091	2.094	2.091
	B	2.791	2.788	2.791	2.788
	C	3.489	3.485	2.791	2.788

Ni-Cr	A	4.470	4.470	3.832	4.470
	B	4.470	4.470	3.832	3.832
	C	4.470	4.470	3.193	3.832

RC	A	0.7024	0.7014	0.7024	0.7016
	B	0.7024	0.7014	0.7024	0.7016
	C	0.7024	0.7014	0.7024	0.7016

*Maximum Von Mises stress in MPa.

**^†^**A—cervical one-third; B—middle one-third; C—apical one-third.

**Table 4:** Comparison of stress* distribution between tapered and parallel posts of different materials with oblique load.

Materials	Region^†^	Tapered post		Parallel post	
		Ferrule	Nonferrule	Ferrule	Nonferrule
Ti	A	5.807	6.403	5.795	6.384
	B	7.258	8.537	7.243	8.512
	C	8.710	8.537	7.243	8.512

Ni-Cr	A	10.24	10.24	10.76	10.94
	B	11.38	11.38	11.95	12.16
	C	10.24	10.24	10.76	10.94

FRC	A	4.195	4.136	4.189	4.133
	B	2.797	3.102	2.793	3.100
	C	6.992	7.236	6.981	8.265

*Maximum Von Mises stress in MPa.

**^†^**A—cervical one-third; B—middle one-third; C—apical one-third.

**Table 5:** Comparison of stress* distribution between tapered and parallel posts of different materials with horizontal load.

Materials	Region^†^	Tapered post		Parallel post	
		Ferrule	Nonferrule	Ferrule	Nonferrule
Ti	A	9.804	10.26	9.802	10.24
	B	11.76	13.19	13.72	13.17
	C	13.72	13.19	11.76	11.71

Ni-Cr	A	14.08	14.07	15.08	15.29
	B	15.65	15.63	16.76	16.99
	C	14.08	14.07	15.08	15.29

FRC	A	5.525	5.626	5.516	5.625
	B	3.684	5.626	3.678	5.626
	C	9.206	11.25	9.192	12.65

*Maximum Von Mises stress in MPa.

**^†^**A—cervical one-third; B—middle one-third; C—apical one-third.

**Table 6:** Comparison of stress* distribution with different posts having ferrule and nonferrule design in the cervical dentin.

Materials	Load^†^	Tapered post		Parallel post	
		Ferrule	Nonferrule	Ferrule	Nonferrule
Ti	V	3.489	3.485	3.489	3.485
	O	18.87	13.87	18.83	13.83
	H	25.49	19.06	25.48	19.02

Ni-Cr	V	2.555	2.555	2.555	2.555
	O	12.52	12.51	11.95	13.36
	H	17.21	17.19	18.43	18.68

FRC	V	3.510	3.505	3.510	3.505
	O	19.57	14.47	19.54	14.46
	H	25.77	19.69	25.73	19.68

*Maximum Von Mises stress in MPa.

**^†^**V—vertical load; O—oblique load (45° angle); H—horizontal load.
